# Zebrafish models for nemaline myopathy reveal a spectrum of nemaline bodies contributing to reduced muscle function

**DOI:** 10.1007/s00401-015-1430-3

**Published:** 2015-05-01

**Authors:** Tamar E. Sztal, Mo Zhao, Caitlin Williams, Viola Oorschot, Adam C. Parslow, Aminah Giousoh, Michaela Yuen, Thomas E. Hall, Adam Costin, Georg Ramm, Phillip I. Bird, Elisabeth M. Busch-Nentwich, Derek L. Stemple, Peter D. Currie, Sandra T. Cooper, Nigel G. Laing, Kristen J. Nowak, Robert J. Bryson-Richardson

**Affiliations:** School of Biological Sciences, Monash University, Melbourne, VIC Australia; Monash Micro-Imaging, Monash University, Melbourne, VIC Australia; Department of Biochemistry and Molecular Biology, Monash University, Melbourne, VIC Australia; Institute for Neuroscience and Muscle Research, The Children’s Hospital at Westmead, Sydney, NSW Australia; Institute for Molecular Bioscience, University of Queensland, Brisbane, QLD Australia; Wellcome Trust Sanger Institute, Wellcome Trust Genome Campus, Hinxton, Cambridgeshire UK; Australian Regenerative Medicine Institute, Monash University, Melbourne, VIC Australia; Discipline of Paediatrics and Child Health, Faculty of Medicine, University of Sydney, Sydney, Australia; Harry Perkins Institute of Medical Research and the Centre for Medical Research, University of Western Australia, Perth, WA Australia

**Keywords:** Nemaline, Myopathy, Zebrafish, Actin, Protein aggregation

## Abstract

Nemaline myopathy is characterized by muscle weakness and the presence of rod-like (nemaline) bodies. The genetic etiology of nemaline myopathy is becoming increasingly understood with mutations in ten genes now known to cause the disease. Despite this, the mechanism by which skeletal muscle weakness occurs remains elusive, with previous studies showing no correlation between the frequency of nemaline bodies and disease severity. To investigate the formation of nemaline bodies and their role in pathogenesis, we generated overexpression and loss-of-function zebrafish models for skeletal muscle α-actin (ACTA1) and nebulin (NEB). We identify three distinct types of nemaline bodies and visualize their formation in vivo, demonstrating these nemaline bodies not only exhibit different subcellular origins, but also have distinct pathological consequences within the skeletal muscle. One subtype is highly dynamic and upon breakdown leads to the accumulation of cytoplasmic actin contributing to muscle weakness. Examination of a Neb-deficient model suggests this mechanism may be common in nemaline myopathy. Another subtype results from a reduction of actin and forms a more stable cytoplasmic body. In contrast, the final type originates at the Z-disk and is associated with myofibrillar disorganization. Analysis of zebrafish and muscle biopsies from ACTA1 nemaline myopathy patients demonstrates that nemaline bodies also possess a different protein signature. In addition, we show that the ACTA1^D286G^ mutation causes impaired actin incorporation and localization in the sarcomere. Together these data provide a novel examination of nemaline body origins and dynamics in vivo and identifies pathological changes that correlate with muscle weakness.

**Electronic supplementary material:**

The online version of this article (doi:10.1007/s00401-015-1430-3) contains supplementary material, which is available to authorized users.

## Introduction

Nemaline myopathy is a congenital skeletal muscle disease characterized by muscle weakness and low muscle tone, except in a few rare patients with hypertonia [20]. Severe nemaline myopathy results in death within the first few months of life; however, individuals displaying milder forms are able to walk despite reduced muscle function [54]. Mutations in ten different genes cause nemaline myopathy: skeletal muscle α-actin (*ACTA1*) [31], nebulin (*NEB*) [40], α-tropomyosin [24], β-tropomyosin [9], troponin T1 [22], cofilin 2 (*CFL2*) [1], *KBTBD13* [45], *KLHL40* [43], *KLHL41* [13] and leiomodin 3 (*LMOD3*) [58]. All of these genes either encode components of the thin filament or regulate thin filament organization and stability. *NEB*, which plays an important role in regulating thin filament length, is the most frequently affected gene in nemaline myopathy with patients exhibiting muscle weakness and reduced force generation [37, 40]. Deletion of exon 55, causing a common form of autosomal recessive nemaline myopathy [26], results in shortened thin filaments, alterations in cross-bridge cycling kinetics, and reduced calcium sensitivity [37, 38].

Nemaline myopathy patients present with a range of clinical phenotypes, but the presence of nemaline (rod-shaped) bodies in skeletal muscles is the defining feature [30, 52]. These structures are thought to derive from the Z-disk and contain Z-disk proteins such as α-actinin [21, 53], although nemaline bodies have also been described in the absence of Z-disk thickening [46]. While nemaline bodies are a hallmark of the disease, their frequency does not correlate with disease severity [3, 17, 28, 41, 42]. In addition, they are sometimes found at the myotendinous junction in healthy skeletal muscle [18, 27] and in healthy ocular muscles [39].

Mutations in *ACTA1* account for ~25 % of nemaline myopathy cases and ~50 % of severe presentations [3]. The majority of *ACTA1* patients carry a single, de novo, dominant mutation but approximately 10 % carry genetic or functional null mutations which are recessively inherited [25]. The dominant ACTA1^D286G^ mutation produces a very severe form of nemaline myopathy [3, 12, 31]. In cell culture experiments, ACTA1^D286G^ expression resulted in the formation of rod-like bodies [7, 49] and the mutant protein demonstrated reduced incorporation into sarcomeric structures [5]. Expression of ACTA1^D286G^ in transgenic mice produced granulofilamentous accumulations in their skeletal muscle. These accumulations stained positive for both phalloidin (labeling F-actin) and α-actinin, and the mice displayed skeletal muscle weakness [41, 42]. Importantly, varying the proportion of mutant actin in this mouse model identified that disease severity correlated with the ratio of mutant to wild-type protein, suggesting a dominant negative action for ACTA1^D286G^ [41].

To investigate the origin of nemaline bodies and to uncover the cause of skeletal muscle weakness, we developed overexpression and loss-of-function zebrafish models for ACTA1-related and a loss-of-function model for NEB-related nemaline myopathy. Remarkably, the in vivo examination of nemaline body formation and progression in *Tg(ACTA1*^*D286G*^-*eGFP)* fish demonstrates that nemaline bodies emanate from the myosepta and are dynamic and transitory in nature. We demonstrate that the breakdown of these early forming nemaline bodies coincides with the formation of globular aggregates that sequester actin-binding proteins and correlates with reduced muscle activity. Conversely, we show that a reduction in α-actin produces a distinct type of Z-disk-derived nemaline body, which extends across the sarcomere, and causes the formation of cytoplasmic α-actinin-rich aggregates. These types of nemaline bodies contribute to impaired muscle function and myofibrillar disarray. From the examination of Neb knockdown fish, we suggest that disruption of α-actin stoichiometry may be a common mechanism of disease for nemaline myopathy. Finally, we also show that the ACTA1^D286G^ mutation has impaired incorporation in the sarcomere, reducing muscle activity, and compounding the effects of the nemaline bodies.

## Materials and methods

### Ethics statement

Fish maintenance and handling were carried out as per standard operating procedures approved by the Monash Animal Services Ethics Committee and the creation of transgenic lines approved by the School of Biological Sciences Animal Ethics Committee (BSCI/2011/18). For patient samples, all experiments were approved by the Children’s Hospital at Westmead Ethics Committee (CHW 2005/042) and the Monash University Human Research Ethics Committee review panel (CF15/743-2015000336).

### Production of transgenic constructs

Zebrafish were maintained as previously described [56]. Transgenic constructs were assembled with the modular tol2 kit [23]. C-terminal eGFP-tagged ACTA1 constructs were created using the following clones: p5E-Bact2, pME-loxP-mCherry-pA-loxP (Genbank accession: KF753698), pME-iCre (Genbank accession: KF753697), pME-ACTA1^D286G^-EGFP, p3E-ACTA1^D286G^-EGFP (Biomatik), p3E-ACTA1^wildtype^ (Biomatik), p5E-actc1b [19], p3E-pA, pDEST-Tol2-pA2 and pDEST-Tol2pA-cryaa:GFP [6]. Transgenic strains generated were *Tg(βAct:loxP*-*mCherry*-*pA*-*loxP:Hs.ACTA1_D286G*-*eGFP)*, *Tg(βAct:loxP*-*mCherry*-*pA*-*loxP:Hs.ACTA1*-*eGFP)* and *Tg(actc1b:iCre)*. Crossing of the *Tg(actc1b:iCre)* strain to either *Tg(βAct:loxP*-*mCherry*-*pA*-*loxP:Hs.ACTA1_D286G*-*eGFP)* or *Tg(βAct:loxP*-*mCherry*-*pA*-*loxP:Hs.ACTA1*-*eGFP)* results in the excision of the *loxP*-*mCherry*-*pA*-*loxP* cassette. The strains generated from this cross are *Tg(βAct:Hs.ACTA1_D286G*-*eGFP* and Tg*(βAct:Hs.ACTA1*-*eGFP*) referred to hereafter as *Tg(ACTA1*^*D286G*^-*eGFP) and Tg(ACTA1*^*wildtype*^-*eGFP)*, respectively. For cardiac actin expression, the *actc1a* ORF was amplified by PCR from IMAGE clone 6893985 (Imagenes), fused to mCherry, and expressed using the *actc1b* promoter [15] cloned into the pDEST-Tol2-pA2 vector.

### cDNA synthesis and quantitative RT-PCR

Total RNA was extracted using TRI Reagent (Sigma). cDNA was synthesized by Superscript III Reverse Transcriptase (Invitrogen Life Technologies). Quantitative RT-PCR (qRT-PCR) was performed using a Lightcycler (Roche) using SYBR Green Master mix (Roche). Primers used for RT-PCR analysis of morpholinos are listed in Supplementary Table 1.

### Gomori trichrome and immunohistochemistry staining

For Gomori trichrome staining on zebrafish tissues, 50-h post-fertilization (hpf) embryos injected with *actc1b*;ACTA1^D286G^-eGFP were anesthetized and snap frozen. Transverse sections (12 μm) were cut using a Leica CM 1850 cryostat. These were imaged for eGFP using a Zeiss Axiophot compound fluorescent microscope. Sections were then stained with modified Gomori trichrome and imaged using a Zeiss Axioskop 2 microscope. Fluorescent and Gomori stained images for the same section were then overlaid for analysis. For immunostaining on zebrafish tissues, 4 % paraformaldehyde (PFA)-fixed 2 dpf whole-mount or 4 dpf vibratome-sectioned embryos were incubated for 10 min in 100 % acetone at −20 °C, washed 3 times in phosphate-buffered saline (PBS) with 0.02 % Tween 20 (PBT), and then incubated for 7 min in trypsin and 3 min in 4 % PFA. Tissues were then washed in PBT and incubated in blocking solution containing the appropriate primary antibody. Antibodies used were anti-α-actinin2 (Sigma clone A7811, 1:100), anti-GFP (Invitrogen A-11122), anti-GFP (DSHB clone 12A6), and AlexaFluor™-labeled-488 and AlexaFluor™-labeled-596 secondary antibodies (Molecular Probes, 1:200). The anti-α-actinin3 antibody was a kind gift of Prof Kathryn North (Murdoch Children’s Research Institute, Melbourne, Australia). The phalloidin used was rhodamine tagged (Molecular Probes, 1:200). Imaging was performed with a LSM 710 confocal microscope (Zeiss), and a 20× 1.0 numerical aperture water-dipping objective for time-lapse experiments or 63× 1.4 numerical aperture oil immersion objective for fixed specimens. All analyses used the ImageJ package Fiji [47].

For nemaline myopathy patient muscle biopsies [18], 8 µm cryosections were collected on glass slides and stained either with Gomori trichrome as described [10] or with anti-α-actinin2 and phalloidin. Immunohistochemistry staining was performed as described [18]. In brief, following air drying, sections were blocked in 2 % BSA–PBS for 15 min before being incubated in blocking solution containing the appropriate primary antibody for 16 h. The anti-α-actinin2 antibody was a kind gift of Prof Alan Beggs (Boston Children’s Hospital, Boston, USA, clone 4B3, diluted 1:1000). The sections were then washed 3 times in PBS and re-blocked for 15 min before being incubated in AlexaFluor™-548 (Molecular Probes, 1:250) and AlexaFluor™-488-phalloidin (Molecular Probes, 1:40) for 1 h. Sections were mounted using Immu-Mount™ (cat #9990402, Thermo Scientific). All sections were imaged using a Zeiss Axioskop 2 microscope.

### Electron microscopy (EM)

For EM 50 hpf, zebrafish embryos were anesthetized and immediately fixed in 2 % PFA/2 % glutaraldehyde in 0.1 M sodium cacodylate buffer (pH 7.5). Samples were post-fixed in 1 % osmium, gradually dehydrated in ethanol, and embedded in Epon epoxy resin using a Pelco Biowave Pro. Ultrathin (90 nm) sagittal sections were cut using an Ultracut UCT ultramicrotome (Leica). For correlative light electron microscopy (CLEM), samples were initially fixed in 4 % paraformaldehyde (PFA)/0.4 % glutaraldehyde in 0.1 M phosphate buffer (pH 7.4) to an equal volume of zebrafish embryo water for 10 min, followed by post-fixation in 2 %PFA/0.2 % glutaraldehyde in 0.1 M phosphate buffer (pH 7.4) overnight at 4 °C. Processing of tissue for ultrathin cryosectioning was carried out as described [35]. For CLEM, the zebrafish were embedded in gelatin blocks and the blocks were infiltrated overnight with 2.3 M sucrose at 4 °C, mounted on aluminum pins, and frozen in liquid nitrogen. Sections were cut on an Ultracut UC7 (Leica) and placed on 50 mesh hexagonal copper grids. The combined immunofluorescence labeling was carried out as described [51] using a biotinylated anti-eGFP antibody (1:300, Rockland #600-106-215), rabbit anti-biotin (1:10.000, Rockland #100-4198), and a goat anti-rabbit AlexaFluor™-488-labeled secondary antibody (Molecular Probes, 1:300). Fluorescence imaging was performed using a Leica AF6000LX Live cell microscope. EM images were taken using an 80 kV Hitachi S-7500 TEM equipped with a Gatan Multiscan digital camera.

### Fluorescence recovery after photobleaching (FRAP) experiments

Two-day-old embryos expressing either ACTA1^D286G^-eGFP or ACTA1^wildtype^-eGFP were anesthetized in 0.16 % tricaine methanesulfonate and laterally mounted in 1 % low melting agarose. Imaging was carried out on a LSM 710 confocal microscope (Zeiss) equipped with a 20× 1.0 numerical aperture water-dipping objective and a 488-nm laser. A precise region of the muscle fiber was bleached with four iterations of 100 % laser and imaged every 20 s for 10 min after bleaching. Four images were taken prior to bleaching and averaged to obtain the pre-bleach value. Fiji [47] was used to determine fluorescence intensity of bleached and unbleached areas at each time point. FRAP data analysis was carried out as previously described [55].

### Morpholino analysis

Morpholino antisense oligonucleotides (MOs) targeted against *nebulin* (exon 5 MO: 5′ TACAGTTCATACCTCACTTAGCTGC, exon 35 MO: 5′ GTCAGTATAGGAATCATACCTGGCT), *actc1b* exon 2 (ex 2) MO (5′ TGCAGTGTTTTTTTCACCTGGTGAC) and *actc1b* 5′ UTR MO (5′ GGTCAAGTTGTTATCACAAGACTGA) (Gene Tools) were diluted in distilled water and co-injected with Cascade Blue-labeled dextran (Molecular Probes) into one- to four-cell embryos at a final concentration of 0.5 μM. The embryos were sorted for Cascade Blue labeling prior to analysis.

### Western blot analysis

100 μg of total protein was extracted from 2 dpf embryos and electrophoresed through a 12.5 % polyacrylamide SDS gel. Protein was transferred to a nitrocellulose membrane and probed with either; anti-α-actin (Sigma clone A2066, 1:400) or β-tubulin (1:5000, Millipore). Secondary HRP antibodies were all used at 1:5000 (Chemicon).

### Swimming assays

Touch-evoked response assays were performed at 2 dpf. Zebrafish larvae were imaged using a high-speed infrared camera (1000 frames per second) following a gentle tap of the head using a blunt needle. The maximum acceleration of the fish was extracted using the ZebraLab software (ViewPoint Life Sciences) with a low detection threshold of 6 mm/s. The *Tg(actc1b*-*eGFP)* zebrafish line was used as a control.

Locomotor activity was examined at 6 dpf by recording the larval activity and swimming distance during a 10-min period using a Zebrabox (ViewPoint Life Sciences). Larvae were gently pipetted into 48-well plates and allowed to habituate in the light for 5 min before the experiment started. Using a high-speed infrared camera (25 frames per second), the embryos were tracked in the dark for 10 min with the following parameters: inactivity threshold of 6 mm/s, detection threshold of 25 mm/s, and activity burst threshold of 30 mm/s. Both the number of small movements (above inactivity threshold and below activity burst threshold) and total distance swum in a 10-min period were extracted using the ZebraLab software (ViewPoint Life Sciences). The *Tg(actc1b*-*eGFP)* zebrafish line was used as a control.

### Statistics

For statistical analysis, a Student’s *t* test assuming equal variance was used to determine significance and performed using Excel (Microsoft) and graphs were prepared in Prism.

## Results

### Overexpression of ACTA1^D286G^-eGFP in zebrafish recapitulates the nemaline myopathy phenotype

**Fig. 1:**
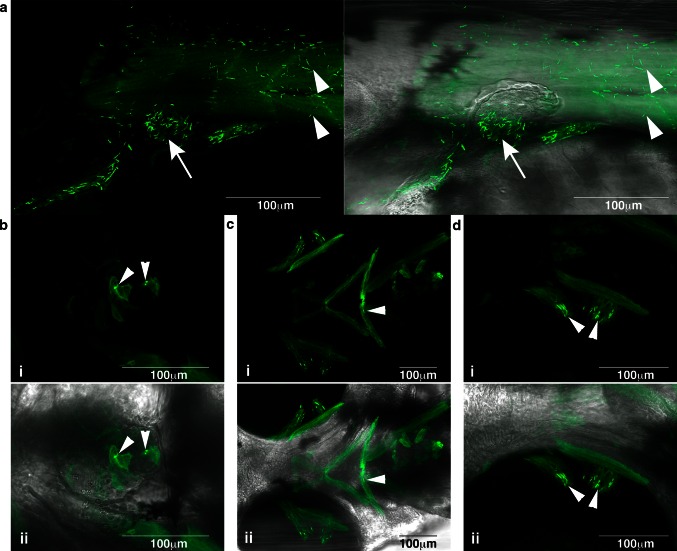
Nemaline bodies form in skeletal muscle in *Tg(ACTA1* ^*D286G*^-*eGFP)*_high_ zebrafish. Nemaline bodies were detected in **a** skeletal muscle (*arrowheads*) and pectoral fins (*arrow*) (*i* and *ii* overlaid with brightfield), **b** heart (*arrowheads*) (*i* and *ii* overlaid with brightfield), **c** facial muscles (*arrowheads*) (*i* and *ii* overlaid with brightfield) and **d** ocular muscles (*arrowheads*; *i* and overlaid with *ii* brightfield)

We created a conditional transgenic zebrafish model for ACTA1 nemaline myopathy, *Tg(ACTA1*^*D286G*^-*eGFP)* that expresses human ACTA1^D286G^ in skeletal muscles. The *Tg(ACTA1*^*D286G*^-*eGFP)*_high_ fish recapitulate hallmark pathological features of the disease with the presence of nemaline bodies in skeletal muscles at 48 and 72 hpf (Supplementary Figure S1). In addition to the trunk musculature, other striated muscles such as the facial, heart, and ocular muscles also contained numerous nemaline bodies (Fig. 1). At 72 hpf, we observed aggregates at the myosepta. These aggregates differ in appearance to the earlier-forming nemaline bodies, having a globular appearance rather than a clear and defined rod shape (Supplementary Figure S1).

**Fig. 2:**
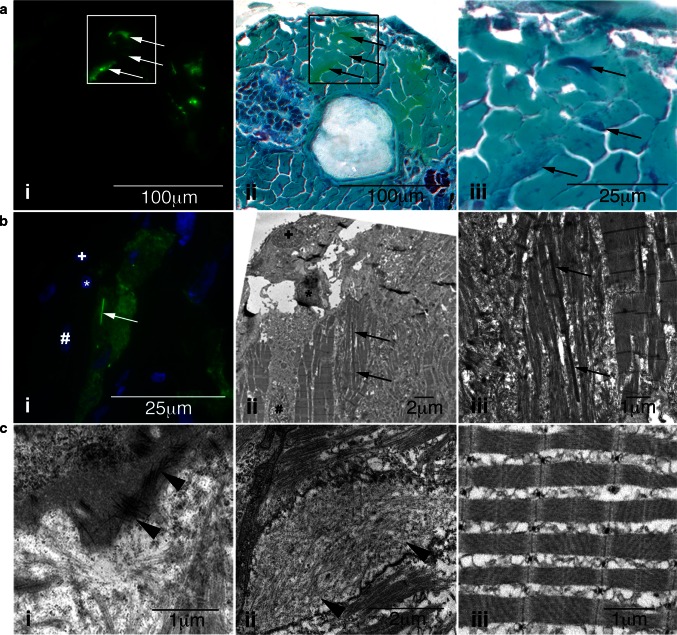
Characterization of skeletal muscle pathology in *Tg(ACTA1* ^*D286G*^-*eGFP)*_high_ zebrafish. **a** Skeletal muscle expressing *i* mosaic ACTA1^D286G^-eGFP, and *ii* overlaid with a light microscopy image of the same section showing Gomori trichrome staining, and *iii* enlarged. *Dark regions* (indicative of nemaline bodies) of disrupted muscle correspond to eGFP expression (*arrows*). **b** Correlative light and electron microscopy of *Tg(ACTA1* ^*D286G*^-*eGFP)*_high_ fish muscle at 2 dpf. **b** *i* Fluorescent image and corresponding *ii* electron microscopy image of skeletal muscle section containing a dense, elongated nemaline body (*arrow*) and enlarged in (*iii*). Sections are matched using nuclei positions (*asterisk*, *plus* and *hash*). **c** *i* Accumulations of actin filaments (*arrowheads*) and *ii* diffuse regions of filamentous actin (*arrowheads*), as well as *ii* disrupted sarcomeric regions are evident in *Tg(ACTA1* ^*D286G*^-*eGFP)*_high_ skeletal muscle, at 2 dpf unlike the *iii* uniform sarcomeres observed in *Tg(ACTA1*-*eGFP)* zebrafish

Gomori trichrome staining of skeletal muscle demonstrated the presence of patches of intense purple staining, indicative of nemaline bodies, correlating with eGFP expression (Fig. 2a). Immuno-CLEM analysis revealed densely stained nemaline bodies that correlated with the eGFP-positive structures observed by confocal microscopy (Fig. 2b). Using conventional electron microscopy on Tg(*ACTA1*^*D286G*^-*eGFP)* zebrafish, we also observed filamentous actin accumulations adjacent to the sarcomere and, occasionally, the sarcomeres appeared disorganized compared to those in *Tg(ACTA1*-*eGFP)* zebrafish (Fig. 2c).

### Nemaline bodies are highly dynamic and transitory in *Tg(ACTA1*^*D286G*^-*eGFP)* fish

**Fig. 3:**
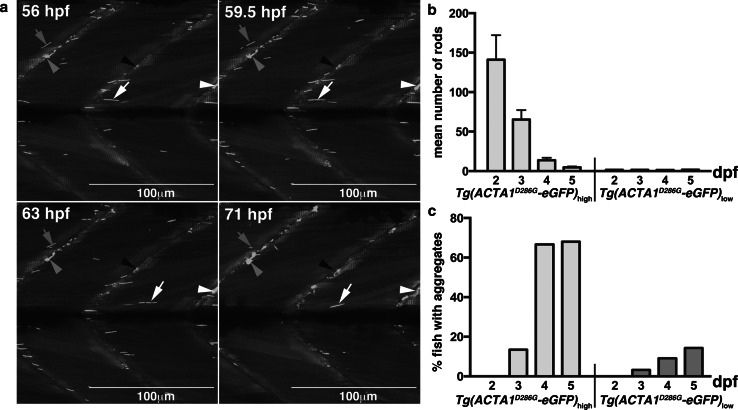
Formation of nemaline bodies and aggregates in *Tg(ACTA1* ^*D286G*^-*eGFP)*_high_ zebrafish. **a** Maximum projection images from time lapse of *Tg(ACTA1* ^*D286G*^-*eGFP)*_high_ fish from 56 to 71 hpf showing nemaline bodies distributed throughout the skeletal muscle (*arrows*). Nemaline bodies’ fragment from 59.5 hpf (*arrows*), coincident with the formation of aggregates at the myosepta (*arrowheads*). **b** Quantification of the mean number of nemaline bodies in *Tg(ACTA1* ^*D286G*^-*eGFP)*_low_ (*n* = 50 per stage) and *Tg(ACTA1* ^*D286G*^-*eGFP)* (*n* = 48 per stage) strains. **c** Quantification of the percentage of fish displaying globular aggregates in *Tg(ACTA1* ^*D286G*^-*eGFP)*_low_ and *Tg(ACTA1* ^*D286G*^-*eGFP)*_high_ strains (*n* = 50 per stage). *Error bars* represent SEM from three independent experiments (*n* = 45 per replicate)

To investigate the origins of the nemaline bodies within the cell, we carried out in vivo time-lapse analysis of the *Tg(ACTA1*^*D286G*^-*eGFP)*_high_ fish. This showed, for the first time, that nemaline bodies form at the sites of muscle attachment at approximately 30 hpf and then extended into the muscle cell (Fig. 3a, Supplementary Movie S1). As development progressed, the nemaline bodies were seen to move throughout the cytoplasm (Fig. 3a, Supplementary Movie S1). The nemaline bodies were highly dynamic, rather than being tethered to the Z-disk or extracellular matrix as previously proposed [46, 57]. By 60 hpf, the characteristic rod-like bodies fragmented and disappeared from the cell, coincident with the formation of the globular aggregates at the myosepta (Fig. 3a, Supplementary Movie S2). Indeed time-lapse analysis of *Tg(ACTA1*^*D286G*^-*eGFP)*_high_ fish showed that as nemaline bodies’ fragment, the fluorescent fusion protein previously incorporated within them can be encapsulated into globular aggregates, at the myosepta (Fig. 3a, Supplementary Movie S2).

**Fig. 4:**
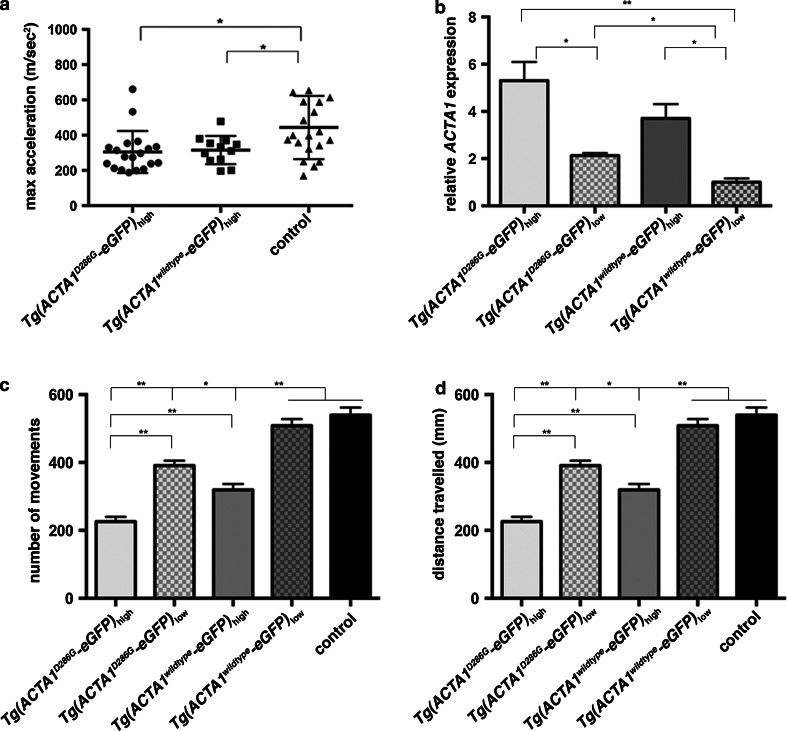
Quantification of muscle function in *Tg(ACTA1*-*eGFP)* zebrafish. **a** Quantification of the maximum acceleration recorded from touch-evoked response assays of *Tg(ACTA1* ^*D286G*^-*eGFP)*_high_ and *Tg(ACTA1* ^*wildtype*^-*eGFP)*_high_ zebrafish compared to control zebrafish at 2 dpf. *Error bars* represent SD for *n* = 15–19 zebrafish, *p < 0.05. **b** qRT-PCR analysis of *ACTA1*-*eGFP* expression in transgenic lines at 2 dpf. No significant difference was observed between *Tg(ACTA1* ^*D286G*^-*eGFP)*_high_ and *Tg(ACTA1* ^*wildtype*^-*eGFP)*_high_ zebrafish. *Error bars* represent ±SEM for four replicate experiments with each experiment comprising a pooled samples of 20 fish, *p < 0.05, **p < 0.01. **c**, **d** Quantification of the **c** number of small movements and **d** distance traveled by *Tg(ACTA1* ^*D286G*^-*eGFP)*_high_ and *Tg(ACTA1* ^*wildtype*^-*eGFP)*_high_ and *Tg(ACTA1* ^*D286G*^-*eGFP)*_low_ and *Tg(ACTA1* ^*wildtype*^-*eGFP)*_low_ strains compared to control fish at 6 dpf. *Error bars* represent ±SEM for three replicate experiments (*n* = 48 per experiment), *p < 0.05, **p < 0.01

In addition to the *Tg(ACTA1*^*D286G*^-*eGFP)*_high_ zebrafish strain that showed high ACTA1^D286G^-eGFP expression, we also constructed a *Tg(ACTA1*^*D286G*^-*eGFP)* low-expressing strain (*Tg(ACTA1*^*D286G*^-*eGFP)*_low_; Fig. 4b). We analyzed the frequency of nemaline bodies during early larval stages in both transgenic strains. We observed almost no nemaline bodies in the *Tg(ACTA1*^*D286G*^-*eGFP)*_low_ strain correlating with the much lower level of ACTA1^D286G^-eGFP expression (Figs. 3b, 4b). Contrastingly, the *Tg(ACTA1*^*D286G*^-*eGFP)*_high_ strain showed the presence of nemaline bodies at 2 dpf, followed by a dramatic reduction at 4 dpf (Fig. 3b). Quantification of the aggregate phenotype identified an increase in the percentage of fish developing globular aggregates in skeletal muscle at 4 dpf, coincident with the disappearance of nemaline bodies (Fig. 3c). This supports the suggestion that early forming nemaline bodies may be one source of the ACTA1 that forms the globular aggregates.

**Fig. 5:**
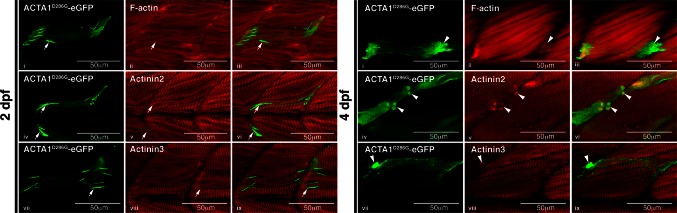
Characterization of nemaline bodies and aggregates in ACTA1-eGFP^D286G^ muscle in zebrafish. At 2 dpf, mosaic expression of ACTA1^D286G^-eGFP in the muscle (*green*) results in the formation of nemaline bodies (*arrows*; *i*, *iv*, *vii*) that do not stain with an actinin2 antibody (*red*; *ii* and overlaid in *iii*), actinin3 antibody (*red*; *v* and overlaid in *vi*), or phalloidin (labeling F-actin, *red*; *viii* and overlaid in *ix*) despite correct localization of these markers in the sarcomere. At 4 dpf, mosaic expression of ACTA1^D286G^-eGFP results in the formation of globular aggregates (*arrowheads*; *i*, *iv*, *vii*) in the muscle (*green*) stain with an actinin2 antibody (*red*; *ii* and overlaid in *iii*), actinin3 antibody (*red*; *v* and overlaid in *vi*) and phalloidin (*red*; *viii* and overlaid in *ix*)

**Fig. 6:**
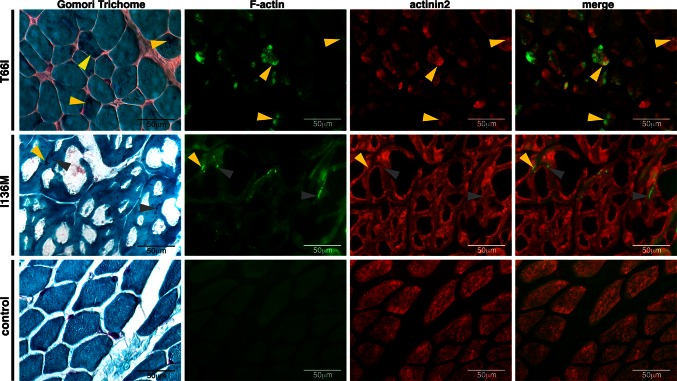
Characterization of nemaline bodies in patient muscle biopsies. In ACTA1^T66I^ muscle, nemaline bodies (*white arrowheads*), darkly stained by Gomori trichome, are positive for actinin2 and phalloidin (labeling F-actin). In ACTA1^I136M^ muscle, nemaline bodies are phalloidin positive and actinin2 positive (*yellow arrowheads*) or actinin2 negative (*gray arrowheads*). Control (unaffected) muscle shows normal localization of markers in the absence of nemaline bodies

To determine if the different aggregates could be distinguished immunologically, we performed antibody and phalloidin (labeling F-actin) staining on zebrafish muscle expressing ACTA1^D286G^-eGFP. Although nemaline bodies could be detected at 2 dpf using an antibody against eGFP, they were not detected by Actinin2 or Actinin3 antibodies, nor by phalloidin (Fig. 5a, Supplementary Figure S2). In contrast, the globular aggregates at 4 dpf were positive for Actinin2, Actinin3 and phalloidin (Fig. 5a, Supplementary Figure S2). We also co-expressed the cardiac α-actin cDNA fused with mCherry (*actc1a*-mCherry) together with ACTA1^D286G^-eGFP in the skeletal muscle and showed that both types of aggregates incorporated cardiac actin (Supplementary Figure S2). To determine if similar α-actinin-negative nemaline bodies were present in patients with mutations in *ACTA1,* we tested patient skeletal muscle biopsies. As observed in *Tg(ACTA1*^*D286G*^-*eGFP)*_high_ zebrafish, we found that nemaline bodies can exhibit different protein signatures. In a biopsy from a patient with a T66I mutation in *ACTA1* [18], nemaline bodies darkly stained by Gomori trichome are both phalloidin and actinin2 positive (Fig. 6). Whereas for a patient with an I136M mutation in *ACTA1* [18], nemaline bodies darkly stained by Gomori trichome are phalloidin positive and either actinin2 positive or negative (Fig. 6).

### Cytoplasmic actin aggregates correlate with reduced skeletal muscle function

Using time-lapse analysis, we observed the formation of globular aggregates in the skeletal muscle and hypothesize that these may contribute to muscle weakness. To test this, we overexpressed a human wild-type *ACTA1* eGFP-tagged transgene in the zebrafish skeletal muscle (Supplementary Figure S1). We constructed transgenic strains with high and low levels of ACTA1^wildtype^-eGFP expression (*Tg(ACTA1*^*wildtype*^-*eGFP)*_high_ and *Tg(ACTA1*^*wildtype*^-*eGFP)*_low_, respectively) (Fig. 4b). In contrast to the rod-shaped nemaline bodies observed in *Tg(ACTA1*^*D286G*^-*eGFP)*_high_ fish, expression of ACTA1^wildtype^-eGFP never induced the formation of rod-like structures (Supplementary Movie S3). Rather, *Tg(ACTA1*^*wildtype*^-*eGFP)*_high_ fish only produced globular aggregates that formed at the myosepta and showed an identical immunological signature to those observed in *Tg(ACTA1*^*D286G*^-*eGFP)*_high_ fish (Fig. 5, Supplementary Figure S2). This demonstrates that globular aggregates are a result of increased ACTA1 and not specifically caused by the *ACTA1*^*D286G*^ mutation. These globular aggregates were formed at earlier developmental stages in *Tg(ACTA1*^*wildtype*^-*eGFP)*_high_ fish (Supplementary Movies S3 and S4) compared to *Tg(ACTA1*^*D286G*^-*eGFP)*_high_ fish (Supplementary Movie S2).

To assess the pathogenicity of globular aggregates in the skeletal muscle, we examined muscle function in both *Tg(ACTA1*^*wildtype*^-*eGFP)* and *Tg(ACTA1*^*D286G*^-*eGFP)* transgenic strains at 2 and 6 dpf. Using a touch-evoked escape response assay, we were able to determine the maximum acceleration of 2 dpf larvae as a direct measure of muscle force. We found a significant decrease in the maximum acceleration of both *Tg(ACTA1*^*wildtype*^-*eGFP)*_high_ and *Tg(ACTA1*^*D286G*^-*eGFP)*_high_ compared to control zebrafish, demonstrating that cytoplasmic actin is pathogenic (Fig. 4a). We also recorded the swimming performance at 6 dpf and observed a significant decrease in the number of movements and in the distance traveled by both *Tg(ACTA1*^*wildtype*^-*eGFP)*_high_ and *Tg(ACTA1*^*D286G*^-*eGFP)*_high_ strains compared to controls (Fig. 4d).

In addition, we assayed the activity of the low-expressing zebrafish strains. While the swimming abilities of *Tg(ACTA1*^*wildtype*^-*eGFP)*_low_ fish were comparable to control fish, *Tg(ACTA1*^*D286G*^-*eGFP)*_low_ fish displayed significantly reduced swimming at 6 dpf (Fig. 4c, d). This reduction in swimming activity correlated with the level of transgene expression (Fig. 4b). These findings demonstrate that cytoplasmic actin aggregates correlate with reduced skeletal muscle performance.

**Fig. 7:**
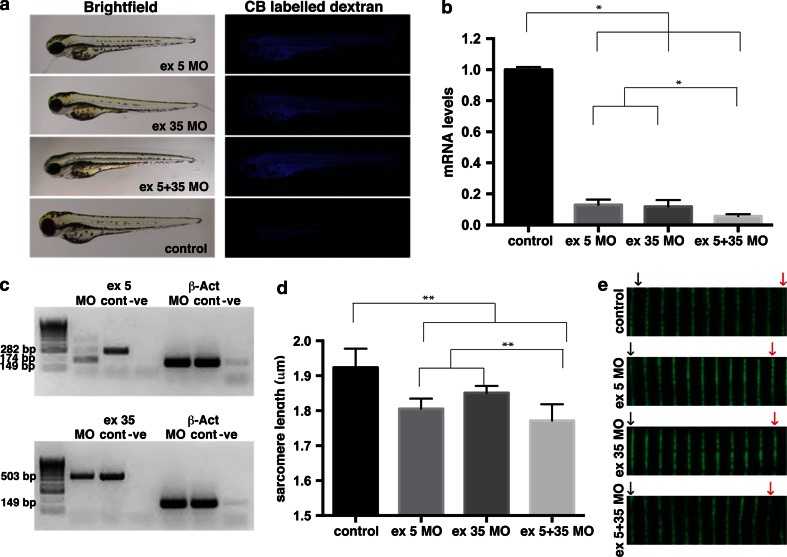
Analysis of nebulin knockdown in zebrafish muscle. **a** Brightfield and fluorescent images of wild-type embryos injected with two different *nebulin* (*neb*)-targeting morpholinos (MOs) compared to control uninjected embryos at 2 dpf. Successful injection is confirmed by the presence of Cascade Blue (CB) labeling. **b** qRT-PCR analysis showing significant knockdown of *neb* transcript in Neb morphants compared to control uninjected (control) at 2 dpf. *Error bars* represent ±SEM for three replicate experiments with each experiment comprising a pooled samples of 20 fish, *p < 0.05. **c** RT-PCR analysis revealed the absence of correctly spliced, and presence of mis-spliced, *neb* transcript in Neb ex5 morphants compared to control uninjected (cont) and reduced expression of *neb* transcript in Neb ex 35 morphants at 2 dpf. β-actin was used as an amplification control. **d** Quantification of sarcomere lengths showed a significant decrease in *neb* morphants compared to controls at 2 dpf. *Error bars* represent ± SEM for four replicate experiments (*n* = 10 per experiment), *p < 0.01. **e** Actinin2 labeling of Z-disks in morphant and control zebrafish at 2 dpf illustrates the reduced sarcomere length in Nebulin morphants compared to controls over 10 sarcomeres (represented by the distance between the *black arrow* and *red arrows*)

**Fig. 8:**
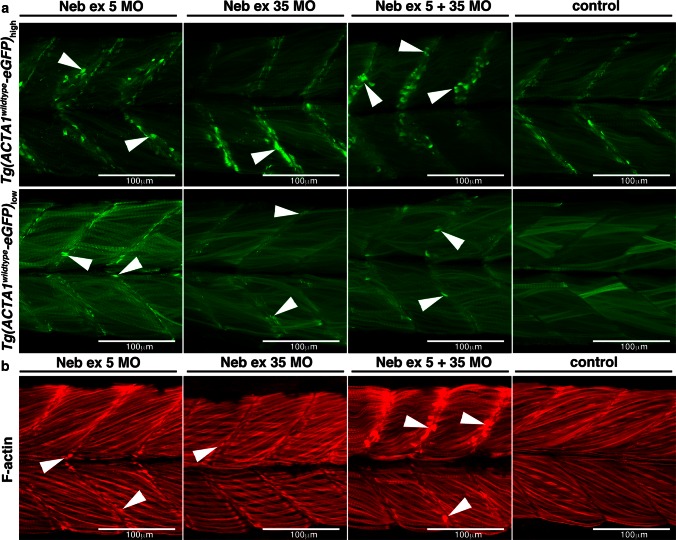
**a** Maximum projection confocal microscopy images of Tg(*ACTA1* ^*wildtype*^-*eGFP*)_low_ and Tg(*ACTA1* ^*wildtype*^-*eGFP*)_high_ zebrafish strains injected with two different Nebulin (Neb) morpholinos compared to control uninjected embryos at 2 dpf. There is an increased prevalence of eGFP-positive globular aggregates at the myosepta (*arrowheads*) in Tg(*ACTA1* ^*wildtype*^-*eGFP*)_high_ Neb morphants compared to controls. Knockdown of Neb produces globular aggregates at the myosepta (*arrowheads*) in Tg(*ACTA1* ^*wildtype*^-*eGFP*)_low_ Neb morphants that are absent in control uninjected embryos. **b** Maximum projection confocal microscopy images of wild-type embryos injected with Neb morpholinos at 2 dpf and stained with phalloidin shows an increase in actin-positive aggregates at the myosepta (*arrowheads*), which are absent in control uninjected embryos

We next examined a model of nemaline myopathy resulting from a loss of Neb to determine whether actin aggregates were a common feature associated with nemaline myopathy. We injected two *neb* splice-site-targeting morpholinos (both singularly and in combination) into wild-type zebrafish. The efficacy of the morpholinos was confirmed by qRT-PCR showing that *neb* mRNA levels are reduced by approximately 80 % by each morpholino and approximately 90 % when both morpholinos are used in combination (Fig. 7b, c). The effect on Neb was further confirmed by examining sarcomere length, with Neb morphants (Fig. 8a) displaying significantly shorter sarcomere lengths compared to controls (Fig. 7d, e).

When *neb* morpholinos were injected singularly and in combination into both the high- and low-expressing *Tg(ACTA1*^*wildtype*^-*eGFP)* strains we observed an increase in eGFP-positive accumulations at the myosepta (Fig. 8a). These accumulations were also phalloidin positive (Fig. 8b), akin to those observed in *Tg(ACTA1*^*D286G*^-*eGFP)*_high_ and *Tg(ACTA1*^*wildtype*^-*eGFP)*_high_ zebrafish, and were not observed in control embryos.

### Loss of α-actin results in Actinin2 accumulation and reduced skeletal muscle function

Our analysis has shown that overexpression of ACTA1^D286G^ produces nemaline bodies and causes detrimental effects on muscle function. However, nemaline bodies have been reported in patients carrying recessive *ACTA1* mutations leading to an absence of skeletal muscle α-actin protein. Skeletal muscle biopsies from these patients contain disorganized myofibrils and nemaline bodies, presumably caused by an imbalanced stoichiometry between sarcomeric components [32].

**Fig. 9:**
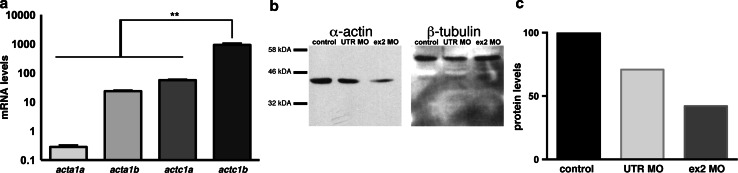
a) qRT-PCR analysis of skeletal α-actin genes in zebrafish tail muscle at 2 dpf. *Error bars* represent ±SEM for three replicate experiments with each experiment comprising a pooled samples of 20 fish, *p < 0.01. **b**, **c** The decrease in total amount of α-actin protein in Actc1b (ex 2 and UTR) morphants at 2 dpf was confirmed and quantitated by Western blot. β-tubulin was used as a loading control

Using our zebrafish system, we also created a model for recessive nemaline myopathy. Zebrafish possess two skeletal muscle α-actin genes (*acta1a* and *acta1b*) and two cardiac α-actin genes (*actc1a* and *actc1b*), which are all expressed during early muscle development. qRT-PCR analyses showed that *actc1b* is the predominant isoform expressed in the skeletal muscle at 2 dpf (Fig. 9a). Thus, we chose to knockdown Actc1b to reduce the amount of α-actin in skeletal muscle.

**Fig. 10:**
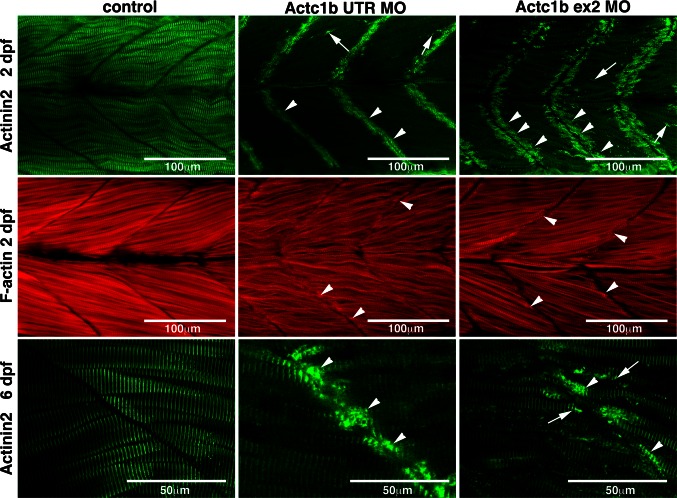
Maximum projection confocal microscopy images of Actc1b morphants and control zebrafish stained with phalloidin at 2 dpf or with an actinin2 antibody at 2 dpf and 6 dpf. Actinin2 and phalloidin-positive nemaline bodies are observed throughout the muscle fibers (*arrows*) as well as projecting from the myosepta (*arrowheads*) in Actc1b morphants compared to controls

**Fig. 11:**
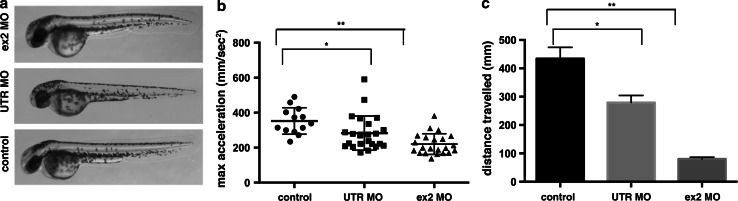
Functional analysis of Actc1b knockdown in zebrafish. **a** Brightfield images of wild-type embryos injected with *actc1b* targeting morpholinos (MO) compared to control uninjected embryos. **b** Quantification of the maximum acceleration recorded from touch-evoked response assays of Actc1b morphants compared to control zebrafish at 2 dpf. *Error bars* represent SEM for three replicate experiments (20 fish per replicate), **p < 0.01. **c** Quantification of the distance traveled by Actc1b morphants compared to control zebrafish at 6 dpf. *Error bars* represent ±SEM for three replicate experiments (*n* = 48 per experiment), **p < 0.01

We injected two morpholinos (one targeting the exon 2 splice donor site and one targeting the 5′UTR). We observed a reduction in α-actin protein by approximately 43 % (Fig. 9b, c) along with decreased phalloidin staining in Actc1b morphants compared to control zebrafish (Fig. 10). Morphologically, Actc1b morphants (Fig. 11a) display impaired skeletal muscle function, showing a reduction in both maximum acceleration at 2 dpf and in swimming distance at 6 dpf (Fig. 11b, c).

**Fig. 12:**
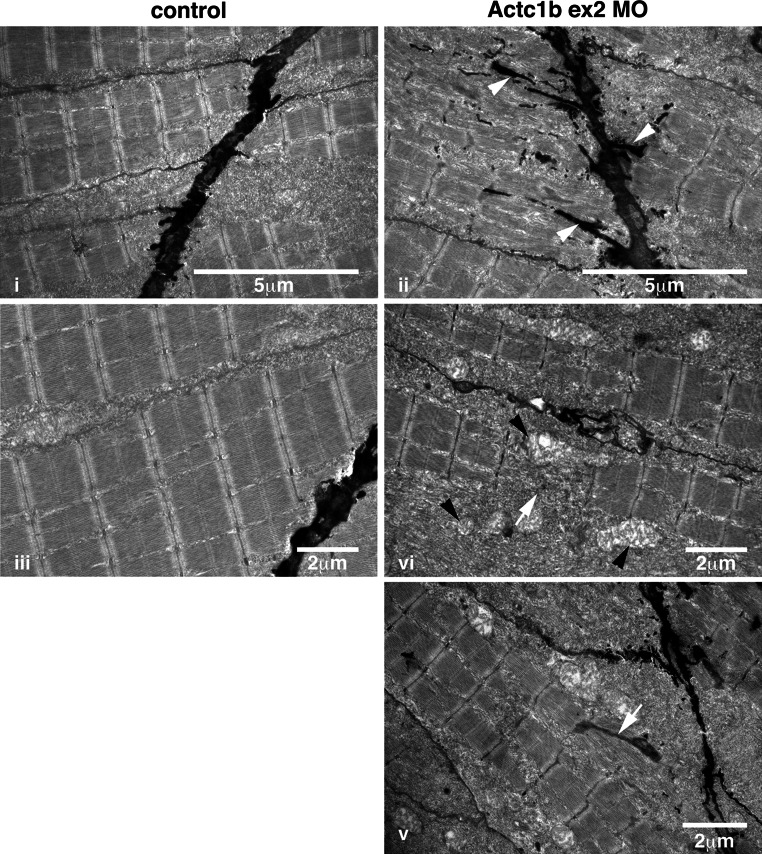
Electron microscopy images of Actc1b ex 2 morphants at 2 dpf showing *ii* electron-dense projections emanating from the myosepta (*arrowheads*) as well as nemaline bodies derived from *v* thickened Z-disks throughout the fibers not observed in control fish (*i*, *iii*). Sections also contained broken muscle fibers *iv* as well as numerous mitochondria (*black arrowheads*; *iv*), compared to the uniform sarcomeres in control zebrafish skeletal muscle *iii*

EM analyses revealed the presence of electron-dense projections extending from the myosepta (Fig. 12). Thickened Z-disks were clearly evident and in some cases the electron-dense region appeared to span the full width of a sarcomere (Fig. 12). We also observed disorganized and broken myofibrils surrounded by large numbers of mitochondria in the Actc1b morphant skeletal muscle compared to control skeletal muscle (Fig. 12). We performed antibody and phalloidin staining on the skeletal muscle and identified phalloidin- and Actinin2-positive nemaline bodies, concentrated near the myosepta, as well as Actinin2-positive aggregates and thickened Z-disks throughout the myofibrils in Actc1b morphants that are not present in the control skeletal muscle (Fig. 10).

We injected the *actc1b* morpholino into the *Tg(ACTA1*^*wildtype*^-*eGFP)*_low_ strain and analyzed the results using in vivo time-lapse imaging. We showed that unlike early forming nemaline bodies in *Tg(ACTA1*^*D286G*^-*eGFP)* muscle, these nemaline bodies were stable in the skeletal muscle, forming from 32 hpf. They were located both at the myosepta and scattered throughout the muscle fibers (Supplementary Movie S5) and were not observed in control embryos (Supplementary Movie S6).

### ACTA1^D286G^ is mislocalized and more rapidly exchanged in the sarcomere

**Fig. 13:**
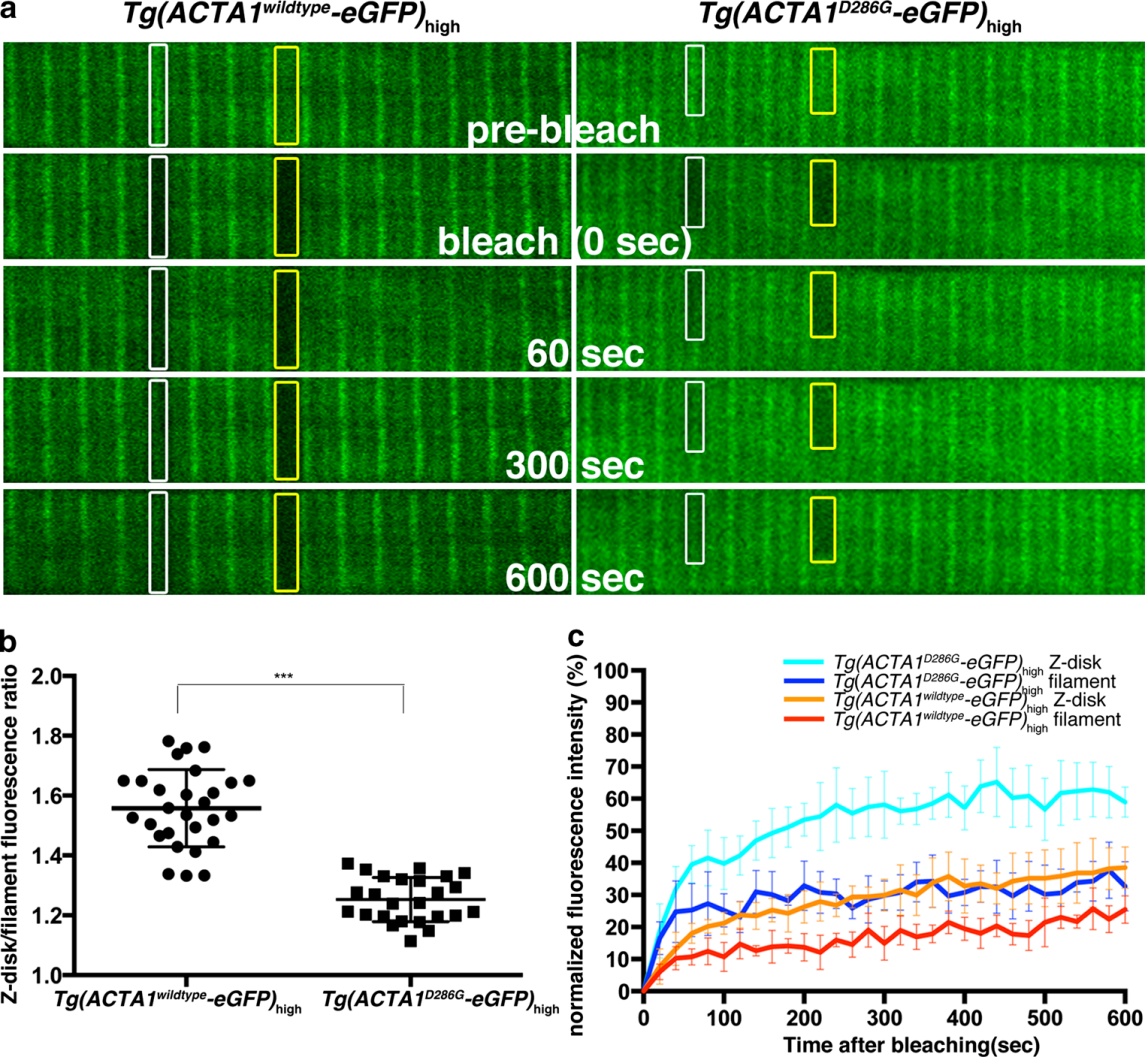
Fluorescence recovery after photobleaching (FRAP) analyses of ACTA1 and ACTA1^D286G^. **a** Confocal images of ACTA1-eGFP localization at the Z-disk (*white boxes*) and along the thin filament (*yellow boxes*) in single muscle fibers of *Tg(ACTA1* ^*D286G*^-*eGFP)*_high_ and *Tg(ACTA1* ^*wildtype*^-*eGFP)*_high_ embryos at 2 dpf. Image sequence shows ACTA1-eGFP prior to photobleaching (pre-bleach), at the time of photobleaching (bleach, 0 s), and 60, 300 and 600 s following photobleaching. Prior to photobleaching eGFP in *Tg(ACTA1* ^*wildtype*^-*eGFP)*_high_ muscle is primarily localized to the Z-disk (*white boxes*), whereas in *Tg(ACTA1* ^*D286G*^-*eGFP)*_high_ fibers, eGFP expression is more diffuse throughout the filament (*yellow boxes*). **b** Quantification of the fluorescence intensity at the Z-disk compared to the filament in *Tg(ACTA1* ^*D286G*^-*eGFP)*_high_ and *Tg(ACTA1* ^*wildtype*^-*eGFP)*_high_ muscle fibers. *Error bars* represent SD for 12 animals (quantifying 2 fibers per animal), ***p < 0.001. **c** Recovery profiles for ACTA1-eGFP and ACTA1^D286G^-eGFP at the Z-disk and filament. *Error bars* represent SD for 8–10 animals (quantifying 2 fibers per animal)

We showed that an imbalance of actin levels in the skeletal muscle results in aggregates and muscle weakness. However, we also wanted to determine whether overexpression of ACTA1-eGFP^D286G^ has an additional, mutation-specific, effect in the skeletal muscle. Previous studies have suggested that ACTA1^D286G^ may have a reduced ability to polymerize and be incorporated into the sarcomere [7, 49]. To examine the dynamics of ACTA1^D286G^ in *Tg(ACTA1*^*D286G*^-*eGFP)*_high_ zebrafish, we performed FRAP analysis on both the thin filament and the Z-disk at 2 dpf. We found a significant reduction in the average time taken for 50 % recovery at both locations following photobleaching for ACTA1^D286G^-eGFP (filament: 22.74 ± 10.22 s, Z-disk: 52.38 ± 9.19 s) compared to *Tg(ACTA1*^*wildtype*^-*eGFP)*_high_ fish (filament: 106.7 ± 34.6 s,: Z-disk: 85.79 ± 16.71 s) (Fig. 13c). This showed that the ACTA1^D286G^ protein is readily incorporated into the sarcomere, but is more rapidly exchanged at both the Z-disk and thin filament than ACTA1^wildtype^, suggesting it may be less stably associated.

During the FRAP analysis, we observed that the localization of the two proteins within the sarcomere differed. While the ACTA1^wildtype^-GFP protein demonstrated a clear striated pattern, with well-defined Z-disk structures, ACTA1^D286G^-eGFP showed more generalized distribution throughout the length of the thin filament (Fig. 13a). We quantified this difference by comparing the fluorescence intensity of the Z-disk to the rest of the filament in both *Tg(ACTA1*^*D286G*^-*eGFP)*_high_ and *Tg(ACTA1*^*wildtype*^-*eGFP)*_high_ fish. We found a significant decrease in the ratio of Z-disk/filament eGFP intensity in *Tg(ACTA1*^*D286G*^-*eGFP)*_high_ (1.3 ± 0.07) compared to Tg(*ACTA1*^*wildtype*^-*eGFP*)_high_ (1.6 ± 0.1) fish. This finding suggested that ACTA1^D286G^ is preferentially localized along the thin filament (Fig. 13b).

## Discussion

We describe the generation of zebrafish ACTA1 nemaline myopathy models allowing us to investigate disease pathogenesis in vivo. Utilizing the advantages of the zebrafish system we analyze, for the first time, nemaline body formation and dynamics in vivo. Through the overexpression of an eGFP-tagged ACTA1 variant or knockdown of α-actin in the skeletal muscle, we observed the formation of three distinct types of nemaline bodies. ACTA1^D286G^ expression resulted in the formation of actinin-negative nemaline bodies at the myosepta, whereas reduction of actin resulted in Z-disk thickening and nemaline bodies within the myofibril and the formation of Actinin2-positive nemaline bodies at the myosepta.

We found that the early forming nemaline bodies in *Tg(ACTA1*^*D286G*^-*eGFP)*_high_ fish are highly dynamic within the cell This is both spatially, with nemaline bodies forming close to the myosepta before moving rapidly throughout the cytoplasm, and temporally, as they form around 48 hpf and degrade by 96 hpf. The transitory nature of the nemaline bodies may explain why there is no correlation between their frequency and disease severity [17, 44]. Following the breakdown of the nemaline bodies, we observe the formation of actin-positive globular aggregates. Swimming behavior at 6 dpf, following the transition from nemaline bodies to globular actin aggregates was impaired, suggesting that cytoplasmic actin accumulation may be detrimental to muscle function.

To test this, we created a line overexpressing the wild-type form of ACTA1. *Tg(ACTA1*^*wildtype*^-*eGFP)*_high_ fish formed globular aggregates, in the absence of nemaline bodies and had reduced muscle performance, indicating that these aggregates likely contribute to reduced skeletal muscle function. Intriguingly, the earlier detection of the aggregates in the *Tg(ACTA1*^*wildtype*^-*eGFP)*_high_ fish rather than in the *Tg(ACTA1*^*D286G*^-*eGFP)*_high_ suggests that the nemaline bodies might delay the formation of these aggregates, most likely through the sequestration of actin. The nemaline bodies may therefore be part of a protective mechanism as suggested for intranuclear rod myopathy [8], and by the observation of rod formation in wild-type muscle under conditions of extreme stress [29].

As our experimental system overexpressed ACTA1, we examined a model of recessive nemaline myopathy to see if similar cytoplasmic actin aggregates were present. Examining Neb morphant embryos, we identified a shortening of the sarcomere length as observed in NEB-deficient animal models [4, 36, 48] and NEB-related nemaline myopathy patients carrying deletions in exon 55 [37]. Neb morphants also displayed accumulations of globular cytoplasmic actin aggregates comparable to those identified in the ACTA1 models, suggesting this may be a common phenotype in the disease, despite a different genetic cause. Recent data have demonstrated an increase in F-actin accumulation, coupled with increased expression of α-actin isoforms in *Cfl2* knockout mice [2, 14] and in patients carrying *CFL2* mutations [1]. Furthermore, it has recently been shown that loss of KLHL40 causes a reduction in NEB, leading to destabilization of the thin filament [11] and increased actin was observed in patients with mutations in *KLHL40* [43]. Together with our data, this suggests that the accumulation of actin in the cytoplasm is one of the contributing factors to reduced muscle performance in nemaline myopathy.

However, the formation of nemaline bodies can not only be caused by the presence of mutant actin, and neither can the accumulation of actin in the cytoplasm be the sole cause of muscle weakness, since loss of ACTA1 also results in the disease [32]. We therefore examined the consequence of reducing actin in the skeletal muscle. We used two different morpholinos to reduce actin levels in the skeletal muscle, both of which resulted in the formation of two further forms of Actinin2-rich nemaline bodies. One originated in the cytoplasm, in the vicinity of the myosepta, and the other appeared to form from Z-disk thickenings that expanded to engulf an entire sarcomere. The cytoplasmic Actinin2-rich aggregates were also positive for actin, suggesting they further deplete the sarcomeric pool of actin, and may impair muscle function in this way. The electron-dense material engulfing the sarcomere would be expected to form a non-contractile region in the fiber reducing function. EM analyses showed that these sarcomeric nemaline bodies were concentrated around regions of fiber disorganization and dissolution, suggesting that their presence interferes with normal sarcomere function causing an inherent structural weakness. This is consistent with a recent analysis of muscle biopsies from NEB-related nemaline myopathy patients that showed myofibrillar dissociation appeared to be an important cause of muscle weakness rather than the frequency or position of nemaline bodies within muscle fibers, which inversely correlated with severity [28]. Functional analysis of Actc1b morphants demonstrated significantly reduced skeletal muscle function at both 2 dpf and 6 dpf, proportional to the loss of α-actin in the skeletal muscle. In Actc1b morphants, we observed electron-dense projections extending from the myosepta, a structure analogous to the myotendinous junction. While disruptions to the myotendinous junction are not thought to be common in patients with ACTA1 mutations, the recessive *ACTA1*^*V154L*^ mutation results in a dystrophic phenotype, alongside Z-disk streaming and infrequent nemaline bodies [33], consistent with dystrophic disruption to this structure.

The frequent presence of Z-disk thickening in nemaline myopathy has led to the prevalent theory that nemaline bodies originate from, and are tethered to, the Z-disk [9, 40, 41, 57]. Our observations show that only one of the three types of nemaline bodies originates within the Z-disk. This is supported by reports of nemaline patients with nemaline bodies but without Z-disk thickening [46] and the observation of nemaline bodies in proximity to the myotendinous junction. The subtypes of nemaline bodies we identified in the zebrafish can be distinguished immunologically. To determine if the same was true in patients, we examined muscle biopsy samples from *ACTA1* nemaline myopathy patients and also found variations in phalloidin and actinin2 labeling of nemaline bodies. In ACTA1^*T66I*^ muscle biopsy samples, nemaline bodies contained both phalloidin and actinin2. However, in ACTA1^*I136M*^, both actinin2-positive and -negative nemaline bodies were identified, confirming previous reports in cell culture [12, 18, 50], and likely corresponding to those identified in the fish. The different composition of the nemaline bodies may allow the subtypes to be used as indicators of the mechanism triggering nemaline body formation and potentially aid in the identification of the causative mutation. Further support for the formation of different types of nemaline bodies comes from examining other nemaline myopathies, such as those caused by a *CFL2* mutation, which have identified a mixture of aggregates, some that stained for F-actin and actinin2, and others that did not [1]. It should be noted that despite the lack of phalloidin labeling, these nemaline bodies in our ACTA1^D286G^ model fish contain actin. The lack of labeling therefore reflects differences in the conformation of actin in the bodies, compared to sarcomeres, preventing binding of phalloidin.

Both our overexpression and loss-of-function ACTA1 nemaline myopathy models show that actin imbalance in the skeletal muscle has pathogenic consequences. In addition to the formation of nemaline bodies and actin aggregates, ACTA1 mutation can also directly affect its sarcomeric function. The ACTA1^D286G^ mutation lies near the hydrophobic pocket, which is important for actin–actin interactions [16]. Due to this location, and the reduced incorporation of ACTA1^D286G^ into actin filaments reported in myoblasts, it has been suggested that the polymerization of ACTA1^D286G^ could be impaired [5]. We directly examined actin dynamics identifying a more rapid exchange of ACTA1^D286G^ compared to ACTA1^wildtype^ protein. Although the mutant actin was incorporated into the sarcomere, we identified a reduced affinity for the Z-disk and increased affinity for the thin filament. These changes in dynamics and sub-sarcomeric localization, coupled with a reduction in binding strength at the actomyosin interface [34], will impact thin filament function in the sarcomere, leading to muscle weakness. Similarly, mutation-specific contributions to muscle weakness are observed in NEB-related nemaline myopathy, where altered thin filament lengths reduce muscle contractile performance [37, 38].

Our study identifies multiple subtypes of nemaline bodies and characterizes, in vivo, their origin within the skeletal muscle. We demonstrate that these subtypes can be differentiated immunologically in skeletal muscle biopsies from ACTA1 nemaline myopathy patients, potentially providing markers for disease severity and aiding in the identification of the causative mutation. We further identify accumulation of cytoplasmic actin, myofibrillar disarray, sarcomeric disruption, and actin depletion as contributors to muscle weakness. Our analysis of a NEB-related nemaline myopathy model and data from other forms of nemaline myopathy suggest that these mechanisms may be common across nemaline myopathy. Furthermore, the generation of these zebrafish models has additionally established an ideal platform for the development and evaluation of therapies.

## Electronic supplementary material

Supplementary material 1 (MP4 2974 kb)

Supplementary material 2 (MP4 12964 kb)

Supplementary material 3 (MP4 3331 kb)

Supplementary material 4 (MP4 6265 kb)

Supplementary material 5 (MP4 6304 kb)

Supplementary material 6 (MP4 3363 kb)

Supplementary material 7 (PDF 8239 kb)
