# Supplementary material for: Zebrafish models for nemaline myopathy reveal a spectrum of nemaline bodies contributing to reduced muscle function
Source: Acta Neuropathol. 2015 May 1;130(3):389–406. doi: 10.1007/s00401-015-1430-3 (PMC4541704; doi:10.1007/s00401-015-1430-3)
Supplement: Supplementary file 7 — Supplementary material 7 (PDF 8239 kb) [file 401_2015_1430_MOESM7_ESM.pdf]

Acta Neuropathologica

Supplementary Information

Zebrafish models for *ACTA1* nemaline myopathy reveal a spectrum of nemaline bodies contributing to reduced muscle function

Tamar E. Sztal[1], Mo Zhao[1], Caitlin Williams[1], Viola Oorschot[2], Adam C. Parslow[1], Aminah Giousoh[3], Michaela Yuen[4], Thomas E. Hall[5], Adam Costin[2], Georg Ramm[2,3], Phillip I. Bird[3], Elisabeth M. Busch-Nentwich[6], Derek L. Stemple[6], Peter D. Currie[7], Sandra T. Cooper[8], Nigel G. Laing[9], Kristen J. Nowak[9], Robert J. Bryson-Richardson[1*].

[1] School of Biological Sciences, Monash University, Melbourne, Victoria, Australia.

[2] Monash Micro-Imaging, Monash University, Melbourne, Victoria, Australia

[3] Department of Biochemistry and Molecular Biology, Monash University, Melbourne, Victoria, Australia

[4] Institute for Neuroscience and Muscle Research, The Children's Hospital at Westmead, Sydney, New South Wales, Australia

[5] Institute for Molecular Bioscience, University of Queensland, Brisbane, Queensland, Australia

[6] Wellcome Trust Sanger Institute, Wellcome Trust Genome Campus Hinxton, United Kingdom

[7] Australian Regenerative Medicine Institute, Monash University, Melbourne, Victoria, Australia

[8] Discipline of Paediatrics and Child Health, Faculty of Medicine, University of Sydney, Australia

[9] Harry Perkins Institute of Medical Research and the Centre for Medical Research, University of Western Australia, Perth, Western Australia, Australia

[*] Corresponding author:

E-mail: robert.bryson-richardson@monash.edu

Telephone: (+61) 3 99024629

Fax: (+61) 3 99055613

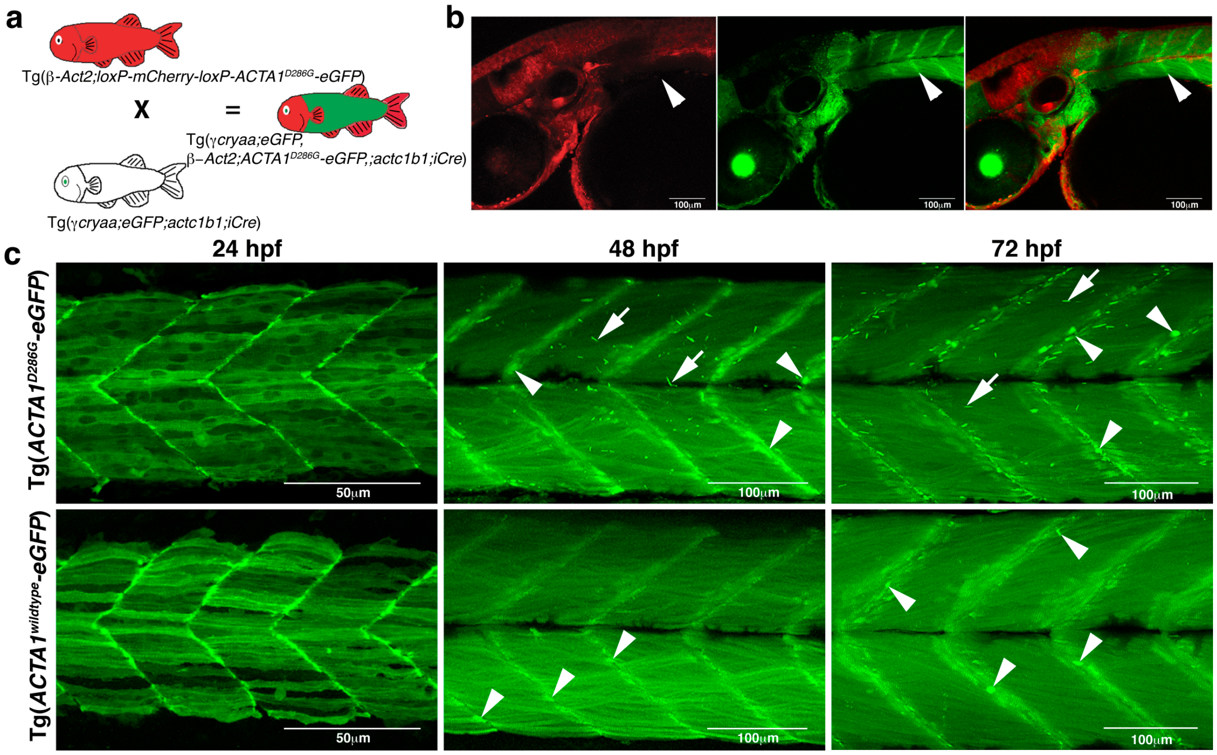

Supplementary Figure S1. Generation and phenotypic characterization of *Tg(ACTA1-eGFP)* stable lines. a) Crossing scheme to generate *Tg(ACTA1-eGFP)* fish. b) Confocal images show that upon iCre-mediated deletion of the loxP cassette, containing mCherry, eGFP is localized to the muscle (green) and does not overlap with other tissues (red; arrowheads). c) Maximum projection confocal microscopy images showing expression of ACTA1-eGFP in zebrafish skeletal muscle at 24, 48, and 72 hpf. $Tg(ACTA1^{D286G}\text{-}eGFP)_{\mathrm{high}}$ expression results in the formation of nemaline bodies (arrows) at 48 hpf and aggregates at the myosepta (arrowheads). No nemaline bodies are observed in $Tg(ACTA1^{wildtype}eGFP)_{\mathrm{high}}$ skeletal muscle at any stage however globular aggregates are evident from 48 hpf at the myosepta (arrowheads).

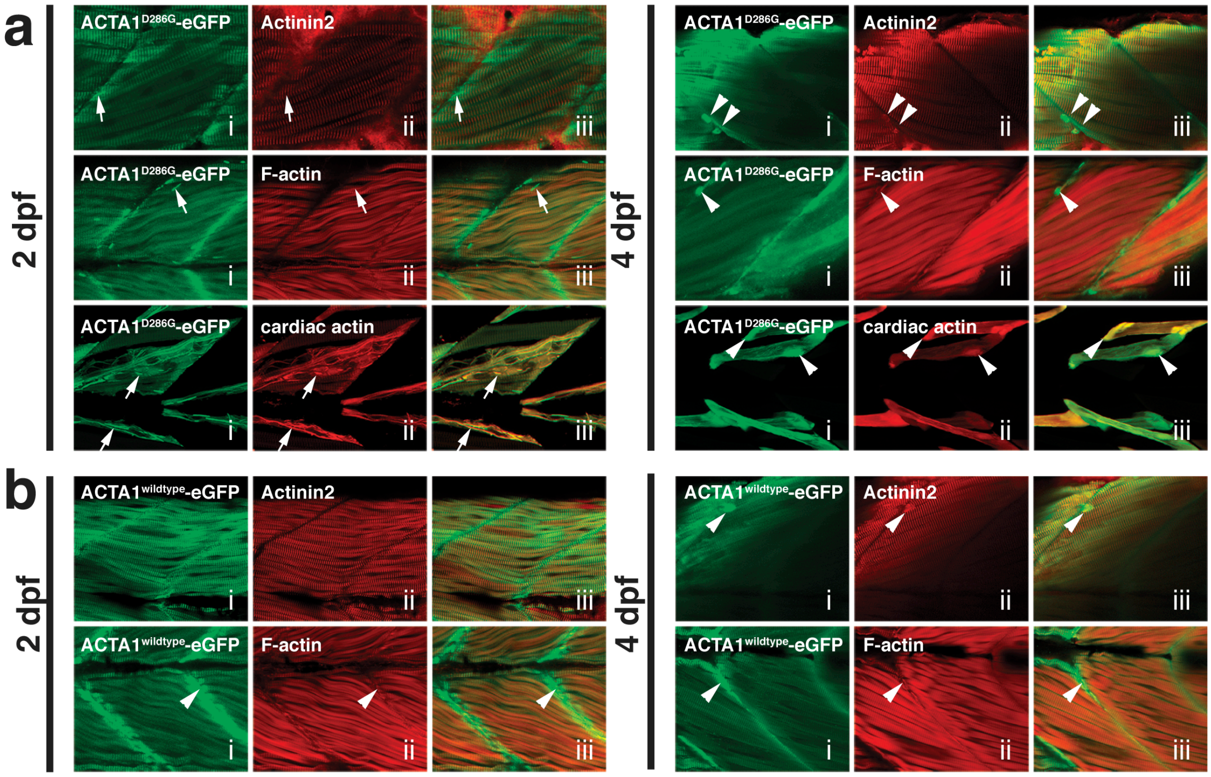

Supplementary Figure S2. Characterization of nemaline bodies and aggregates in *Tg(ACTA1-eGFP)*$_{\text{high}}$ zebrafish. a) Confocal microscopy images of nemaline bodies and aggregates in *Tg(ACTA1*^{D286G}*-eGFP)*$_{\text{high}}$ muscle. At 2 dpf, nemaline bodies (arrows) in (i) *Tg(ACTA1*^{D286G}*-eGFP)*$_{\text{high}}$ muscle (green) do not stain with Actinin2 (red; ii and overlaid in iii) or phalloidin (labeling F-actin) (red; i and overlaid in ii) despite correct localization of these markers in the sarcomere. At 4 dpf, aggregates (arrowheads) in (i) *Tg(ACTA1*^{D286G}*-eGFP)*$_{\text{high}}$ muscle (green) stain for Actinin2 (red; ii and overlaid in iii), and phalloidin (red; ii and overlaid in iii) in *Tg(ACTA1*^{D286G}*-eGFP)*$_{\text{high}}$ muscle (green). Co-injection of *actc1b*;ACTA1$^{\text{D286G}}$-eGFP and *actc1b*;*actc1a*-mCherry (cardiac α-actin) results in mosaic expression of both constructs throughout the skeletal muscle. ACTA1$^{\text{D286G}}$-eGFP (green) and cardiac actin (red) co-localize in nemaline bodies at 2 dpf (arrows; ii and overlaid in iii) and in aggregates at 4 dpf in (arrowheads; ii and overlaid in iii). b) Confocal microscopy images of globular aggregates in *Tg(ACTA1*^{wildtype}*-eGFP)* $_{\text{high}}$ muscle. At 2 dpf (arrows), and 4 dpf, aggregates (arrowheads) are observed in (i) *Tg(ACTA1*^{wildtype}*-eGFP)*$_{\text{high}}$ skeletal muscle (green) labeled with Actinin2 (red; ii and overlaid in iii) and phalloidin (red; ii and overlaid in iii).

Supplementary Movie S1. Time-lapse movie showing a lateral view of eGFP labeled muscle in *Tg(ACTA1^{D286G}-eGFP)*$_{high}$ fish from 30 hpf, showing nemaline bodies forming at the myosepta (arrows). Each frame is a maximum projection of a confocal image series. Anterior is to the left. Interval between frames is 30 minutes. Frame rate is 7 frames per second.

Supplementary Movie S2. Time-lapse movie showing lateral view of eGFP labeled muscle in *Tg(ACTA1^{D286G}-eGFP)*$_{high}$ from 54 hpf, showing nemaline bodies moving throughout the cytoplasm and fragmenting coincident with the formation of globular aggregates at the myosepta. Each frame is a maximum projection image of a confocal image series. Anterior is to the left. Interval between frames is 30 minutes. Frame rate is 7 frames per second.

Supplementary Movie S3. Time-lapse movie showing lateral view of eGFP labeled muscle in *Tg(ACTA1^{wildtype}-eGFP)*$_{high}$ fish from 30 hpf. Each frame is a maximum projection of a confocal image series. Anterior is to the left. Interval between frames is 10 minutes. Frame rate is 7 frames per second.

Supplementary Movie S4. Time-lapse movie showing zoomed in lateral view of eGFP labeled muscle in *Tg(ACTA1^{wildtype}-eGFP)*$_{high}$ fish from 54 hpf showing globular aggregates forming at the myosepta. Each frame is a maximum projection of a confocal image series. Anterior is to the left. Interval between frames is 30 minutes. Frame rate is 7 frames per second.

Supplementary Movie S5. Time-lapse movie showing lateral view of eGFP labeled muscle in *Tg(ACTA1^{wildtype}-eGFP)*$_{low}$ fish injected with an Actc1b morpholino from 31 hpf. Nemaline bodies originate from within muscle fibres as well as projecting from the myosepta. Each frame is a maximum projection of a confocal image series. Anterior is to the left. Interval between frames is 10 minutes. Frame rate is 7 frames per second.

Supplementary Movie S6. Time-lapse movie showing lateral view of eGFP labeled muscle

in *Tg(ACTA1^wildtype-eGFP)*$_{low}$ fish from 31 hpf. Each frame is a maximum projection of a confocal image series. Anterior is to the left. Interval between frames is 10 minutes. Frame rate is 7 frames per second.

Supplementary Table 1: Primers sequences used for analysis of morpholinos and qRT-PCR

Gene	Primer Name	Primer sequence	Orientation
nebulin	exon 5 MO	TGGTGGTCTTCCTAGGTGCT	forward
nebulin	exon 5 MO	TCACGAGCCTCACCATCATA	reverse
nebulin	exon 35 MO	TGAGCACAACTACCGCACTC	forward
nebulin	exon 35 MO	GAACCTTTGAGGCCATTTTG	reverse
β-actin	RTF	GCATTGCTGACCGTATGCAG	forward
β-actin	RTR	GATCCACATCTGCTGGAAGGTGG	reverse
ACTA1	ACTA1-eGFP	GAGCGCAAATACTCGGTGTG	forward
ACTA1	ACTA1-eGFP	GAACAGCTCCTCGCCCTTG	reverse
nebulin	RTF	CAAATACAGGGAGGAATATGAAA	forward
nebulin	RTR	CCTTTTTGATGCGAATCATCT	reverse
acta1a	RTF	AAAGCAGAGGACGGGTTTGT	forward
acta1a	RTR	CTTGATGGGTTCTGCCTCTGCTCGT	reverse
acta1b	RTF	ATTCATCGGCTGCATCTGTC	forward
acta1b	RTR	CCCAAAGCTGTCCCATAATTT	reverse
actc1a	RTF	ATCTTCAATCCTTCTCTGTTCAGTC	forward
actc1a	RTR	TATACAGCACAAGGCACAGTACAA	reverse
actc1b	RTF	TCCCAGTGGAAGCATCAGTC	forward
actc1b	RTR	TGGGGTACTTCAGGGTCAAG	reverse
